# Extra-adrenal, non-functional adrenocortical carcinoma presenting with acute abdomen: a case report

**DOI:** 10.1186/s13256-020-02408-7

**Published:** 2020-07-08

**Authors:** Alireza Mirsharifi, Mohammad Vasei, Ehsan Sadeghian, Ali Ghorbani-Abdehgah, Sara Naybandi Atashi

**Affiliations:** 1 Department of General Surgery, Research Center of Surgical Outcomes and Procedures, Shariati Hospital, Tehran University of Medical Sciences, Tehran, Iran; 2 Cell-Based Therapies Research Center, Digestive Disease Research Institute, Shariati Hospital, Tehran University of Medical Sciences, Tehran, Iran; 3 Department of Radiology, Shariati Hospital, Tehran University of Medical Sciences, Tehran, Iran

**Keywords:** Adrenocortical carcinoma, Children, Mitotane, Acute abdomen

## Abstract

**Background:**

Adrenocortical carcinoma is a rare malignancy, with 43% being non-functional. These may arise from adrenal rest anywhere in the embryonic pathway of the adrenal glands. In the context of extra-adrenal and retroperitoneal tumors, the exact pathologic diagnosis is challenging. The case reported here, to the best of our knowledge, is the seventh reported case of extra-adrenal non-functional adrenocortical carcinoma.

**Case presentation:**

We report a case of extra-adrenal non-functional adrenocortical carcinoma in a 15-year-old Persian boy who presented with an acute abdomen. He underwent surgical resection. Pathologic findings based on immunohistochemistry and cellular morphology confirmed adrenocortical carcinoma. He was treated with mitotane for 24 months. During a follow-up period of 30 months, no recurrence or metastases were found.

**Conclusion:**

Despite the rarity of extra-adrenal adrenocortical carcinoma, presentation with an acute abdomen may occur, and the tumor may be found anywhere in the adrenal embryonic pathway. On the other hand, tumor behavior and prognosis in children may be different from what we expect in adults.

## Introduction

Adrenocortical carcinoma (ACC) is a rare malignancy with an annual incidence of 1–2 in one million [1, 2]. Various hormone oversecretions can be detected in 40–60% of ACCs, leading to a wide range of clinical presentations [1].

Functional tumors account for 57% of these tumors and non-functional tumors make up the other 43% [2]. In non-functional tumors, 79% of the cases are reported to be symptomatic. These symptoms are usually vague chronic abdominal discomfort. Asymptomatic cases are found incidentally during abdominal imaging or unrelated surgery [1]. Although most of the tumors arise from the adrenal glands, there are also rare cases which arise from the adrenal rest. Most of the adrenal rest tumors are thought to be functional [3]. Non-functional adrenal rest tumors are rare [3]. Adrenal rest may be found in any anatomical site along the embryonic pathway. Patients with this ectopic ACC usually present with a mass effect, which is caused by tumor overgrowth, necrosis, or hemorrhage.

Extra-adrenal non-functional ACC is an extremely rare tumor with only six cases having been reported to the best of our knowledge [3–8], and here we present the seventh case. We try to explain the different aspects of this kind of tumor, from pathology and diagnosis, to management and prognosis.

This case is the first reported case of extra-adrenal carcinoma, which presented with an acute abdomen, making the diagnosis before laparotomy very difficult.

## Case presentation

Our patient was a 15-year-old Persian boy who was referred with an acute-onset right lower quadrant pain. His symptoms started with a gradually worsening generalized abdominal pain for 24 hours. Other symptoms were anorexia, nausea, and vomiting. He had no remarkable past medical, familial, and habitual history. He did not take any medication previously. He was a student in the fourth grade of middle school.

On examination he had tachycardia (pulse rate of 115 beats per minute) but all other vital sign parameters were normal. An abdominal examination revealed severe generalized abdominal tenderness with rebound tenderness and abdominal wall guarding. These findings proved acute abdominal syndrome which necessitated emergent laparotomy.

Ultrasonography reported a hypoechoic region adjacent to his gall bladder extending down to the lower pole of his right kidney suggesting fluid collection. Intra-abdominal fluid and a few lymph nodes in the right lower quadrant with the greatest dimension of 12 mm were also detected.

Laboratory tests revealed neutrophilic leukocytosis (white blood cells of 23000/mm^3^) but electrolytes, blood glucose, urea levels, and coagulation tests were found to be in the normal range. An upright chest X-ray was normal without free air.

He underwent emergency midline laparotomy with the diagnosis of an acute abdominal syndrome with suspicion of a complicated acute appendicitis. During a complete exploration, a circumscribed rubbery mass (measuring 8 cm in greatest diameter) was detected in the retroperitoneum at the medial side of the right kidney, with no local invasion or adhesion. The mass had been ruptured, and large amounts of bloody fluid were found in his abdominal cavity. The rest of the abdominal cavity exploration was negative. The tumor measured 8 cm in greatest diameter; it was resected completely.

Histopathologic examination revealed a neoplasm in which the neoplastic cells were arranged in sheets. There were medium to large polygonal cells with well-defined faint eosinophilic cytoplasm and round nuclei. Also, there were cells with clear cytoplasm. The stroma contained a rich network of capillaries with foci of necrosis and hemorrhage. In immunohistochemistry (IHC) staining, the cells were diffusely positive for steroidogenic factor 1 (SF-1), synaptophysin, and Melan A, focally for α-inhibin, and scattered S100 staining, but were negative for cytokeratin (CK) AE1/AE3, chromogranin, and human melanoma black 45 (HMB45) (Table 1). The results of histopathology and IHC were consistent with ACC.

**Table 1:** Immunohistochemistry findings

Marker	Interpretation
Pan cytokeratin AE1/AE3	Negative
SF-1 (steroidogenic factor 1)	Diffusely positive
Synaptophysin	Diffusely positive
Alpha inhibin	Focally positive
Melan A	Diffusely positive
PAX8 (paired-box gene)	Negative
EMA (epithelial membrane antigen)	Negative
Chromogranin	Negative
S100 protein	Positive in a few tumor cells
CD10	Negative
Vimentin	Negative
Calretinin	Positive
Ki-67 protein	6%
HMB45 (human melanoma black 45)	negative

In postoperative metastasis workup, serum metanephrine level, urine catecholamine, dexamethasone suppression test, aldosterone level, and renin activity were all within normal limits.

**Fig. 1:**
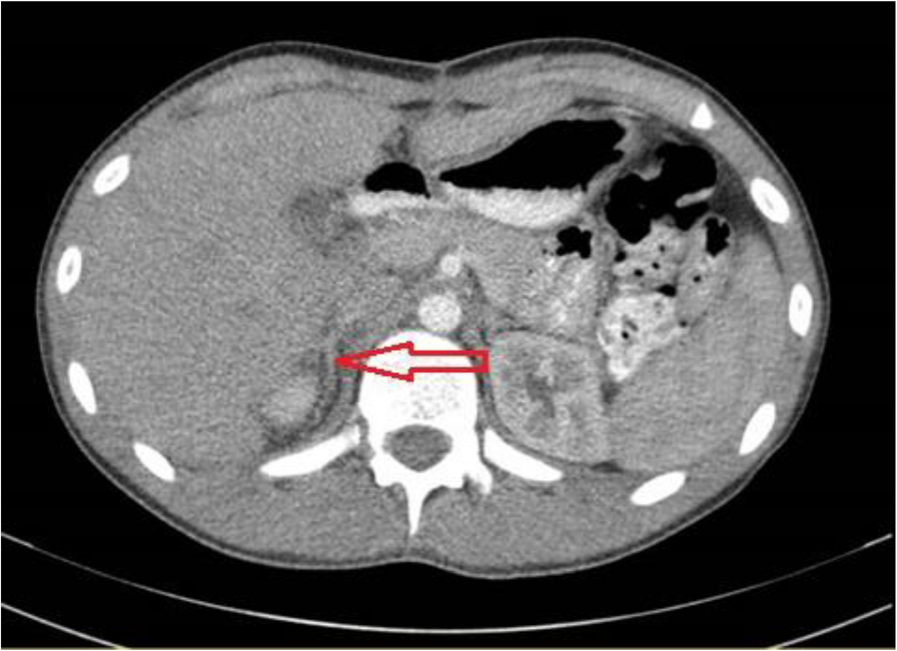
Normal appearance of adrenal glands is evident in postoperation computed tomography scan shown with *arrow* (after tumor resection and before adrenalectomy)

A computed tomography (CT) scan of his chest, abdomen, and pelvis with intravenously administered contrast revealed no adrenal mass or significant lymphadenopathy. Normal appearing right adrenal gland visualized in Fig. 1. Hyperparathyroidism was ruled out by neck ultrasonography and by normal serum calcium, phosphorous, and parathyroid hormone level. Following a multidisciplinary board meeting, 2 weeks after the first laparotomy, our patient underwent right adrenalectomy through a right subcostal incision (anterior approach). Pathology reported normal adrenal tissue with mild hyperplasia in the adrenal medulla. He was discharged with no unexpected event on postoperative day 4.

Mitotane (3 g/day divided dose) was started 3 weeks after the operation and continued for 24 months.

Our patient continues to be disease-free after a follow up of 30 months, with no significant clinical signs or symptoms. He had monitoring CT scans at 6, 12, and 24 months after surgery. The CT scans did not show any evidence of recurrence. Since there were no signs of hormonal oversecretion, we did not perform any additional hormonal laboratory testing for follow up.

## Discussion

ACC is a rare malignancy with an annual incidence of 1–2 in one million [1, 2]. We presented the case of a 15-year-old boy with an odd presentation of extra-adrenal non-functional ACC. Although this type of tumor has been reported before (Table 2), the presentation with acute abdominal pain makes it unusual. He underwent surgical resection of the tumor and right adrenalectomy. Mitotane was administered for 24 months. In follow up he had no recurrence in 30 months.

**Table 2:** Extra-adrenal non-functional adrenocortical carcinomas

Author(s) and reference number	Age (year) and sex	Size	Location	Management	Follow up
Current case	15 M	8 cm	In retroperitoneum at the medial side of the right kidney	Resection followed by mitotane	NED at 30 m
Cornejo *et al*. [5]	51 M	10 cm	Pelvic mass arising within the soft tissue between the prostate and bladder	Resection followed by mitotane and RT	NED at 9 m
Bani-Hani [4]	52 M	19 cm	Mass lying between spleen, stomach, tail of pancreas, splenic flexure and left kidney and adrenal gland	Resection	NED at 25 m
Rodriguez *et al*. [6]	0.4 F	6 cm	Spinal cord at T10–L2	Laminectomy with gross total resection	LR at 6 m s/p second gross total resection, undergoing chemotherapy (cisplatin, doxorubicin, etoposide, mitotane) 5 m postoperation
Goren *et al.* [3]	50 F	8 cm	Hilum of left kidney	Radical nephrectomy	NED at 12 m
Yokoyama *et al*. [7]	34 F	6.5 cm	Retroperitoneum	Retroperitoneal mass between right kidney and IVC resection	Resection NED at 120 m
Lee *et al*. [8]	61 M	13 cm	Right intrarenal	Adjuvant chemotherapy (VAP; vincristine, doxorubicin, and prednisolone) with mitotane	NED at 3 m

*F* female, *IVC* inferior vena cava, *LR* local recurrence, *M* male, *m* month, *NED* no evidence of disease, *RT* radiotherapy, *s/p* status post

The diffuse area of necrosis and hemorrhage, and the presence of more than 5% positive cells for Ki-67 immunostaining suggest that our case was more probably an ACC and not an adenoma. Adrenal adenocarcinoma is a rare diagnosis and in 40–60% of cases, elevated adrenocortical steroid hormones are detected [1]. In non-functional tumors, abdominal discomfort and pain is the most frequent symptom, although almost 20% of non-functional tumors are asymptomatic. Almost always, ACCs arise from the adrenal gland, but, rarely, they may arise from an adrenal rest [5, 7]. Adrenal rest may be found in any anatomical site along the embryonic pathway including celiac axis, genitalia, and broad ligaments [9].

The etiology of ACCs is not clear but it seems p53 mutation has a role in children’s ACCs. This mutation may predict cases with familial and carrier cases [10]. On the other hand, transactivation region (human immunodeficiency) ribonucleic acid (TAR (HIV) RNA) binding protein 2 (*TARBP2*) gene overexpression seems to be a discriminating factor between ACCs and adenomas [11].

Abdominal pain due to tumor growth may happen, but acute abdomen is a very rare presentation in ACC, although there are a few cases that reported acute abdomen as the presentation of pheochromocytoma [12–14]. ACC cases reported with acute abdomen seem much less frequent [15, 16]. As far as we are aware, there are no reported cases of extra-adrenal ACC, which presented with an acute abdomen. This presentation is thought to be due to necrosis and hemorrhage or a ruptured tumor [13, 16]. Differential diagnoses of the masses in retroperitoneum in pediatric patients are soft tissue sarcomas, Wilms tumor arising from a kidney, or neuroblastoma.

The majority of ACCs can be differentiated from adenoma; however, a subset of cases may present a true diagnostic challenge. Weiss proposed prognostic criteria based on the analysis of certain microscopic features, such as nuclear anaplasia, mitotic rate, diffuse architecture, necrosis, capsular invasion, venous invasion, and sinusoidal invasion. Some authors claimed that these criteria are not fully applicable in adrenocortical tumors of children [17]. The 5-year survival rate of ACC is reported to be between 20 and 35% [18].

**Fig. 2:**
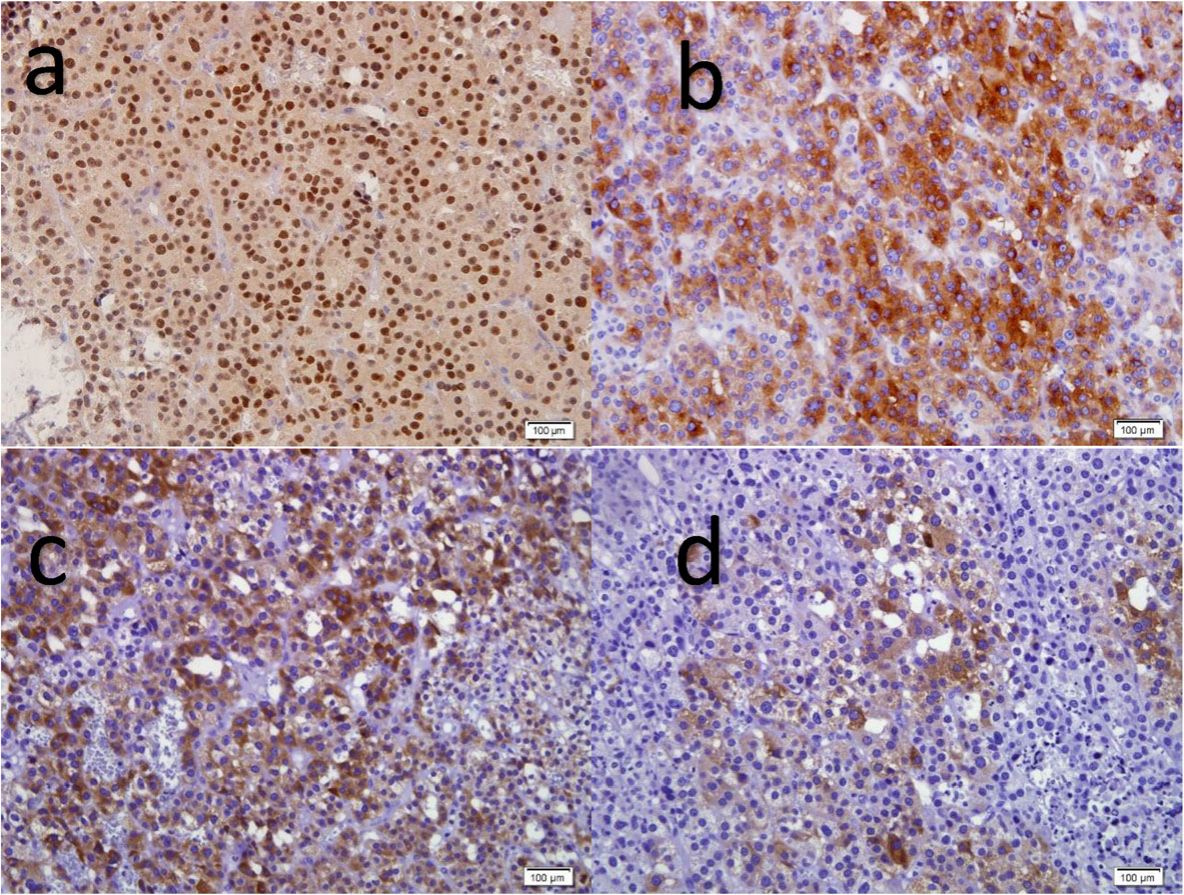
Immunohistochemistry for steroidogenic factor 1 (**a**) and synaptophysin (**b**) as well as Melan A (**c**) shows diffuse nuclear and cytoplasmic positive reactions respectively. α-inhibin positivity was focal (**d**) 3,3'-diaminobenzidine staining × 400

Some correlation exists between the microscopic degree of differentiation, proliferative activity measured by counting mitoses, or assessing Ki-67 positivity and survival [19]. Proliferation rate as determined by Ki-67, or mitotic count > 5/50 high-power field (HPF), in combination with necrosis have been validated as highly predictive of the outcome in carcinomas [20, 21] (Fig. 2).

In a study by Wieneke and colleagues on 83 patients under 20 years of age, 74 cases were identified as carcinoma based on conventional criteria; whereas, in 23 patients, the malignant clinical behavior of a tumor was seen [22]. For this reason, a new scoring system was proposed for predicting the behavior of tumors in pediatric patients. Wieneke *et al.* concluded that vena caval invasion, necrosis, and increased mitotic activity independently suggest malignant features in patients under 20 [22]. Our case’s tumor was low grade in histology regarding nuclear morphology, as well as low Ki-67 index, and showed a favorable outcome in a 30-month postoperative period concordantly.

Mitotane can be used as an adjuvant treatment for patients who have a high risk of recurrence. High-risk tumors are defined as Ki-67 of more than 10% or R1 resection. It is important to be aware of the side effects of mitotane, which mostly target digestive and neurologic systems and adrenal insufficiency [1].

Although mitotane was administered, our patient’s long disease-free period may be related to the less aggressive behavior of the tumor rather than to the adjuvant therapy.

## Conclusion

ACCs may present with an acute abdomen. This tumor may be found anywhere in the adrenal embryonic pathway. Although it has been suggested to start mitotane for cases with lymph node metastasis, it seems to be rational to prescribe it in ruptured ACCs even with no lymph node involvement or in extra-adrenal ACCs. Malignant features of adrenocortical tumors in histopathologic findings should be interpreted cautiously in pediatric tumors, because adult malignancy criteria may not be applicable in children.

## Data Availability

Data sharing is not applicable to this article as no datasets were generated or analyzed during the current study. Para clinic data which is referred to in the case presentation is available on request from the corresponding author.

## Abbreviations

ACC: Adrenocortical carcinoma
IHC: Immunohistochemistry
CT: Computed tomography
